# Supplementary material for: Mapping the human praxis network: an investigation of white matter disconnection in limb apraxia of gesture production
Source: Brain Commun. 2022 Jan 13;4(1):fcac004. doi: 10.1093/braincomms/fcac004 (PMC8833454; doi:10.1093/braincomms/fcac004)
Supplement: fcac004_Supplementary_Data [file fcac004_supplementary_data.pdf]

Hannah Rosenzopf; Daniel Wiesen; Alexandra Basilakos; Grigori Yourganov; Leonardo Bonilha; Christopher Rorden; Julius Fridriksson; Hans-Otto Karnath; Christoph Sperber

Mapping the human praxis network: an investigation of white matte disconnection in apraxia of gesture production

Supplementary materials

1 Distribution of ABA-2 Limb Apraxia Scores

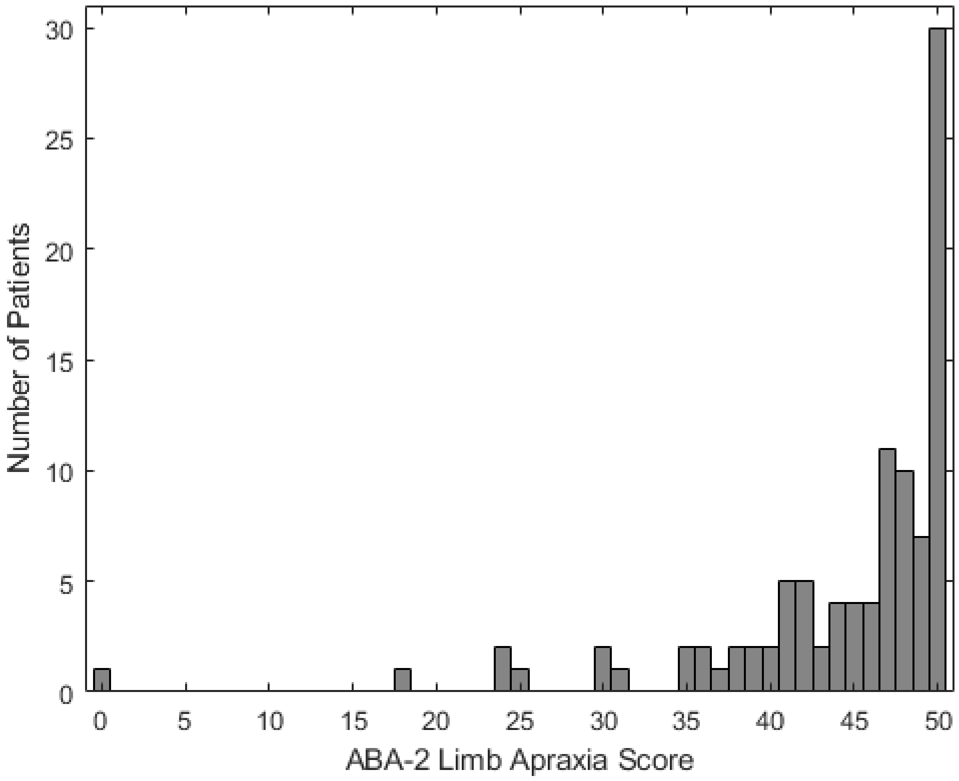

Supplementary Figure 1: Distribution of ABA-2 Scores for all 101 patients.

2 Overlap topographical results with a probabilistic cytoarchitectonic atlas

Supplementary Table 1: Overlap between fibre tracts according to the Juelich probabilistic cytoarchitectonic atlas (Bürgel *et al.* 2006) and the voxels found to be significant in our analysis. Fibre tracts with less than 20mm³ overlap are not depicted.

Affected fibers	Number of affected voxels/mm³
Acoustic radiation	68
Callosal body	94
Corticospinal tract	779
Inferior occipitofrontal fascicle	37
Optic radiation	39

3 Region-based evaluation of disconnectivity - Percentage of disconnected streamlines

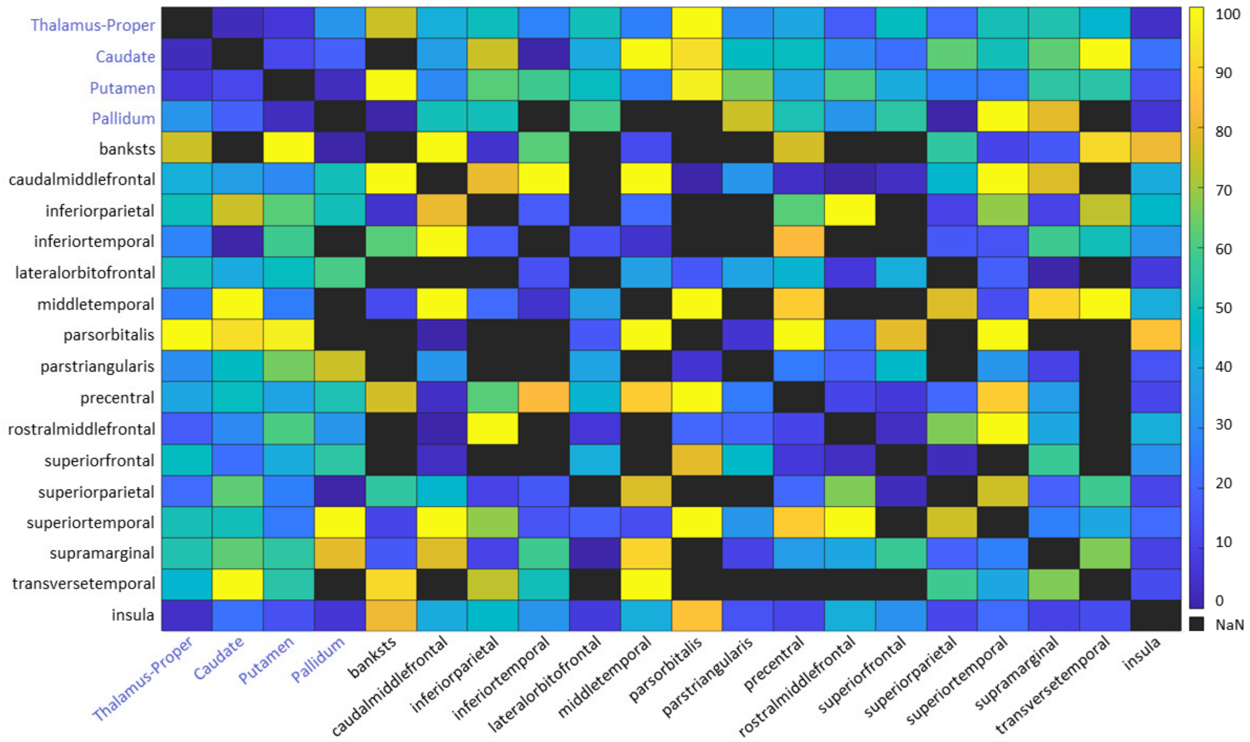

Supplementary Figure 2: Region-based evaluation of disconnectivity
Heatmap of the 20 areas with the highest absolute disconnection as shown in Figure 4, but showing relative disconnection here. Cell colour refers to the percentage of disconnected streamlines.

4 Additional SVR-LSM analysis with non-linear kernel

As an additional analysis, we mapped the structural lesion anatomy underlying apraxia. We replicated the methods of the FA-SVR mapping on the binary lesion maps, including a linear kernel, hyperparameter optimisation to find a trade-off between model fit and reproducibility, and false discovery rate correction after 25.000 permutations. As reported in the main paper, no significant results remained after false discovery rate correction.

This null result might be rooted in the inability of linear kernels to model the information given by focal lesion data. Non-linear kernels might be better suited to model anatomo-behavioural data (see Hope et al., 2018). However, feature weights cannot be computed in non-linear kernels, and, therefore, SVR-lesion symptom mapping (SVR-LSM; Zhang et al., 2014) is, strictly speaking, not possible. In the current study, we utilised a linear kernel in the SVR-FA mapping for this reason. However, it was shown that, with a specific

algorithm for lesion size control, SVR-LSM can be computed with non-linear kernels if we accept certain approximations (Zhang et al., 2014).

Therefore, we additionally analysed the structural lesion data with a non-linear kernel. Hyperparameters were optimized using an approach proposed by Mirman (https://github.com/dmirman/SVR-LSM/blob/master/functions/optimize_parameters.m). The script, which automatically maximizes model fit (regardless of reproducibility) achieved R^2 = 0.37 with an optimized c=3, and an optimized γ=0.5 as ideal hyperparameters. We ran SVR-LSM controlled for lesion size with direct total volume control (dTLVC) and correction by false discovery rate and referenced significant voxels to the same grey matter brain atlas that we used in the regional disconnectivity analysis in the main paper (Desikan et al., 2006). The majority of significant voxels (7516 out of 13550) could not be assigned to any grey matter atlas region, reflecting a high percentage of white matter contribution. Supplementary Table 2 shows the areas with the highest number of significant voxels in descending order. A complete table of all atlas areas can be found in the online materials.

Supplementary Table 2: Results of the SVR-LSM with non-linear kernel. Atlas areas were defined by the brain atlas by Desikan and colleagues (2006). All affected structures were located in the left hemisphere.

Atlas area	Absolute number of significant voxels	Equivalent loss of area (in %)
putamen	1434	22.0
inferiortemporal	772	4.6
temporalpole	617	13.9
middletemporal	580	3.0
lateralorbitofrontal	516	3.9
pallidum	489	35.0
amygdala	404	13.5
fusiform	206	1.0
superiortemporal	195	1.0
insula	190	1.4

5 Overview tractogram creation

Evaluation of structural disconnection was assessed by generating a white matter link-wise disconnectome. The goal was to assess which connections between region of interest (ROI)

pairs were altered due to white matter disruption found in the analysis. Grey matter ROIs were defined according to the Desikan-Killiany atlas and its 84 ROIs retrieved from the IIT Human Brain Atlas (v.5.0) (https://www.nitrc.org/projects/iit/ ; [Zhang & Arfanakis, 2018]). Disconnections were quantified for the statistical map resulting from the SVR-Fa-mapping analysis shown in Figure 2. The creation of the tractogram and the connectome were conducted in MRtrix3 (https://www.mrtrix.org/; Tournier et al., 2012). We used the Spherical harmonic (SH) coefficients of the IIT_HARDI.nii template file (Varentsova et al., 2014) from the IIT Human Brain Atlas (v 5.0.) as input to perform tractography. To prepare the mask for streamline seeding we started with the command *5tt2gmwmi* on the IIT_fornix_fixed_5tt_file_for_ACT_tractography.nii, available from the IIT Human Brain Atlas (v.5.0). The mentioned template contains a 5 tissue type segmented anatomical image, which enabled us to anatomically constrain the streamlines and hence, to improve the accuracy of streamline termination (Smith *et al.*, 2012). The *tckgen* command was used to generate streamlines. To do so, we used the default probabilistic algorithm (*iFOD2*, Tournier *et al.*, 2010) with anatomical constrained deconvolution. 10 million streamlines were seeded at the grey matter – white matter boundary. Additionally, *backtrack* (Smith *et al*., 2012) allowed to resample streamlines that had been rejected previously, in case of a poor structural termination. Next, *tcksift* was used. The command executes filtering of tracks with the so-called spherical-deconvolution informed algorithm (Smith *et al.*, 2013). This transforms the streamline densities to match the FOD lobe integrals and reduces overestimation of longer tracks. Secondly, it turns the number of streamlines between two regions into a proportional estimate of the cross-sectional area of fibres that connect the two concerned regions. Consecutively, the connectome of the resulting whole-brain tractogram was created with *tck2connectome*, by quantifying the number of streamlines between any two Desikan-Kiliany ROIs (Desikan *et al.,* 2006). Additionally, we created a second connectome file for our SVR-FA-mapping statistical result by removing first all streamlines of the whole-brain tractogram running through the statistical map with *tckedit*. Then, we again quantified the number of streamlines in a same way as for the 'healthy' connectome and took the difference between both. This allows us to specifically quantify which areas of the brain are structurally disconnected or altered (i.e. the disconnectome) with reference to our statistical findings.

Additional References

Bürgel U, Amunts K, Hoemke L, Mohlberg H, Gilsbach JM, Zilles K. White matter fiber tracts of the human brain: Three-dimensional mapping at microscopic resolution, topography and intersubject variability. Neuroimage 2006; 29: 1092–1105.

Desikan RS, Ségonne F, Fischl B, Quinn BT, Dickerson BC, Blacker D, et al. An automated labeling system for subdividing the human cerebral cortex on MRI scans into gyral based regions of interest. Neuroimage 2006; 31: 968–980.

Hope, T. M. H., Leff, A. P., & Price, C. J. (2018). Predicting language outcomes after stroke: Is structural disconnection a useful predictor? NeuroImage: Clinical, 19(March), 22–29. https://doi.org/10.1016/j.nicl.2018.03.037

Smith, R. E.; Tournier, J.-D.; Calamante, F. & Connelly, A. Anatomically-constrained tractography: Improved diffusion MRI streamlines tractography through effective use of anatomical information. NeuroImage 2012; 62: 1924-1938

Smith RE, Tournier, JD, Calamante F, Connelly A. SIFT: Spherical-deconvolution informed filtering of tractograms. NeuroImage, 2013; 67: 298-312

Tournier JD, Calamante F Connelly A. Improved probabilistic streamlines tractography by 2nd order integration over fibre orientation distributions. Proc ISMRM 2010; 1670.

Varentsova A, Zhang S, Arfanakis K. Development of a high angular resolution diffusion imaging human brain template. NeuroImage 2014; 91: 177–186.

Zhang S, Arfanakis K. Evaluation of standardized and study-specific diffusion tensor imaging templates of the adult human brain: Template characteristics, spatial normalization accuracy, and detection of small inter-group FA differences. NeuroImage 2018; 172: 40-50.
